# Evaluation of the Toxicity and Toxicokinetics of Cereulide from an Emetic *Bacillus cereus* Strain of Milk Origin

**DOI:** 10.3390/toxins8060156

**Published:** 2016-06-06

**Authors:** Yifang Cui, Yuan Liu, Xiaoye Liu, Xi Xia, Shuangyang Ding, Kui Zhu

**Affiliations:** 1 Beijing Advanced Innovation Center for Food Nutrition and Human Health, College of Veterinary Medicine, China Agricultural University, Beijing 100193, China; yayapi19@163.com (Y.C.); liu_yuan@cau.edu.cn (Y.L.); leaf19880705@126.com (X.L.); xxia@cau.edu.cn (X.X.); 2 Department of Biomedical Engineering, Duke University, Durham, NC 27708, USA

**Keywords:** *Bacillus cereus*, cereulide, toxicity, toxicokinetics, UPLC-MS/MS

## Abstract

*Bacillus cereus* is an opportunistic foodborne agent causing food poisoning and many infectious diseases. The heat-stable emetic toxin cereulide is one of the most prevalent toxins produced by pathogenic *B. cereus*, resulting in symptoms such as emesis and liver failure. In the present work, the toxicity and toxicokinetics of cereulide from an emetic *B. cereus* isolate (CAU45) of raw milk were evaluated. The production of cereulide was tested by a cytotoxicity test and enzyme immunoassay, and confirmed by the presence of the *ces* (cereulide synthetase) gene and the ultra-performance liquid chromatography tandem mass spectrometry (UPLC-MS/MS) method. All results showed that the amount and toxicity of cereulide produced by CAU45 was 7 to 15.3 folds higher than the reference emetic *B. cereus* DSMZ 4312. Cereulide in plasma was collected at different time points after a single intravenous injection to evaluate its toxicokinetics in rabbits. The maximum concentration of cereulide was achieved in 2.6 ± 3.4 h after administration, with the elimination half-life of 10.8 ± 9.1 h, which expands our understanding of the toxic effects of cereulide. Together, it suggests that urgent sanitary practices are needed to eliminate emetic toxins and emetic *B. cereus* in raw milk.

## 1. Introduction

*B. cereus* is a spore-forming, ubiquitously opportunistic pathogen, causing various diseases including food poisoning, fulminant bacteremia, pneumonia and endophthalmitis [1]. Foodborne *B. cereus* causes two types of symptoms: diarrhea and emesis [2]. Two tripartite enterotoxin complexes, non-hemolytic enterotoxin (Nhe) and hemolysin BL (Hbl), and a single component of cytotoxin K1 (CytK1) are potentially responsible for diarrhea [3,4], whereas emetic syndrome is induced by a lipophilic cyclic dodecadepsipeptide cereulide, which previously formed in the contaminated foods. Additionally, cereulide has been reported to result in fulminate liver failure in children after ingestion of *B. cereus*-contaminated spaghetti and pesto [5,6].

*B. cereus* can be found in most common food matrices such as rice, pasta and dairy products, and it represents more than 68.0% of foodborne outbreaks in all food types [7]. There is an increasing occurrence of *B. cereus* foodborne outbreaks. For example, *B. cereus* was responsible for 13.4% of bacterial foodborne outbreaks, ranking as the second among the most frequent bacterial causes of foodborne disease in Chinese inland provinces [8]. In fact, the number of *B. cereus* contaminations is highly underestimated, because foodborne *B. cereus* disease is mild and self-limiting and is consequently not reported by patients. The presence of cereulide or emetic *B. cereus* in foods is a severe hazard for consumer safety. The heat-stable, proteolytically resistant and preformed cereulide is still active and can cause intoxication after the heat treatment in food processing and passing through the intestinal tract [9]. For instance, emetic *B. cereus* strains have been detected not only in farinaceous foods commonly correlated with cereulide intoxication but also in cheese, salads and meat products [10]. *B. cereus sensu stricto* isolates have also been shown to produce Nhe and Hbl enterotoxins in milk and dairy products in China [11,12]; however, the presence of cereulide in milk or related products has not been reported yet.

Cereulide is synthesized by non-ribosomal peptide synthetases (NRPS) encoded by the *ces* gene cluster [13]. As an ionophore with high affinity to K^+^, cereulide can interfere the electrochemical potential gradient on lipid membranes and inhibit mitochondrial activity [14,15]. Although cereulide has been shown to induce emesis in animal models [16,17], the toxicokinetics of cereulide remain unclear. In this work, we firstly characterized an emetic *B. cereus* isolate from raw milk in a local dairy farm of Beijing, China, based on the presence of the *ces* gene and a marker protein of cereulide (CBP) by immunoassay. Secondly, we quantified the production of cereulide by UPLC-MS/MS, and evaluated its virulence with a cytotoxicity test. Lastly, the toxicokinetics of cereulide were evaluated in rabbits.

## 2. Results

### 2.1. Characterization of B. cereus CAU45

**Figure 1:**
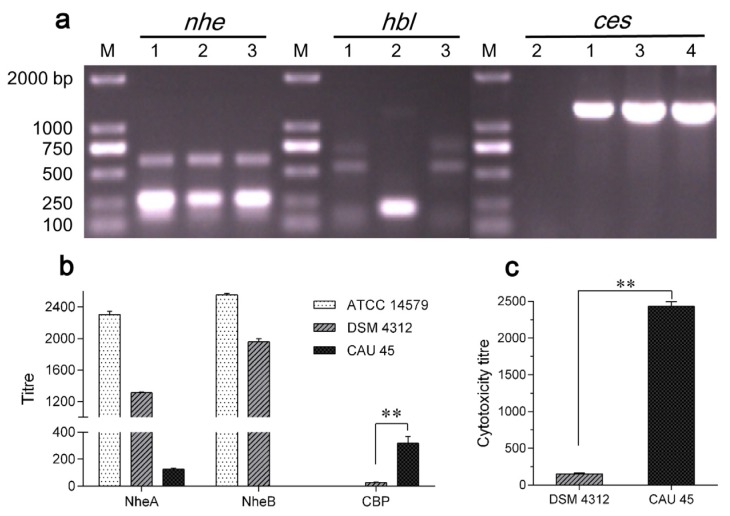

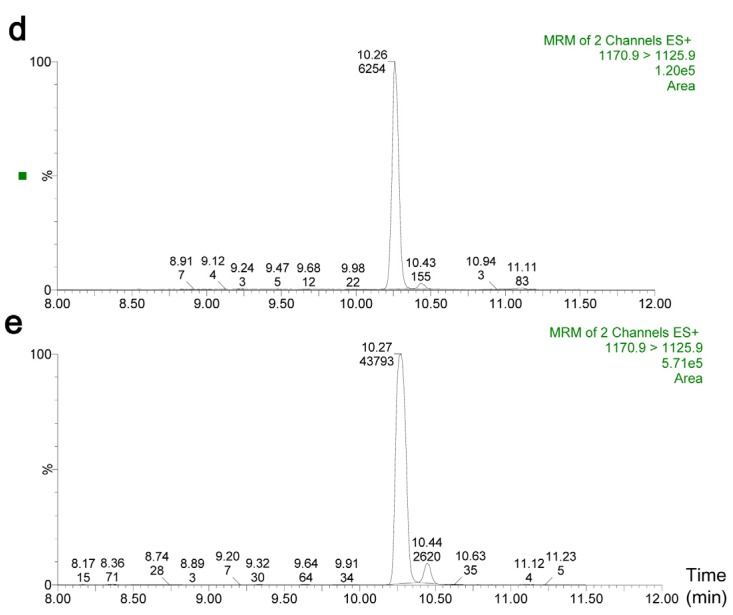
Characterization of the toxin profiles of raw milk isolated *B. cereus* CAU45 strain. (**a**) Identification of the toxin genes *nhe*, *hbl* and *ces* by PCR assays. The presence of *nheA*, *nheB* and *nheC*, *hblA* and *hblD*, and *ces* was tested. The amplicons of targets were sequenced and submitted for sequence analysis. Lanes 1–4 were the reference strains of *B. cereus* DSMZ 4312 containing *nhe* and *ces* genes (1), *B. cereus* ATCC 14579 containing *nhe* and *hbl* genes (2), and raw milk isolated *B. cereus* CAU45 (3) and its plasmid (4). M, size marker (TransGen Biotech, Beijing, China); (**b**) The presence of NheA, NheB, and maker protein of cereulide (CBP) was detected based on specific monoclonal antibodies. For *B. cereus* CAU45, low titer of NheA was detected, and no NheB was found. The presence of CBP was found in *B. cereus* DSMZ 4312 and CAU45 strains, and showed significant difference (*n* = 3, ** *P* = 0.0097); (**c**) Cytotoxicity of cereulide was tested in HEp-2 cells. Means ± SD were presented throughout (*n* = 3, ** *P* = 0.0003); (**d**,**e**) UPLC chromatograms of cereulide in *B. cereus* DSMZ 4312 (**d**) and CAU45 strains (**e**).

The *B. cereus* CAU45 strain was tested for the presence of four main toxin genes, *nhe*, *hbl*, *cytK1* and *ces*. The presence of *nheA*, *nheB* and *nheC*, and *ces* genes was found in *B. cereus* CAU45 and confirmed by sequence analysis, as shown in Figure 1a, and the *ces* gene cluster was located on a plasmid of *B. cereus* CAU45. Subsequently, the ability of Nhe and cereulide production was tested by immunoassays and cytotoxicity tests. A low level of NheA and no NheB was detected by monoclonal antibodies (mAbs) 1A8 and 1E11 in *B. cereus* CAU45, respectively (Figure 1b). However, the titer of CBP in *B. cereus* CAU45 (270 ± 15) was 8.7 folds higher than that in *B. cereus* DSMZ 4312 (31 ± 4) (Figure 1b, *P* = 0.0097). This was consistent with the cytotoxicity result that the cytotoxic titer of cereulide from *B. cereus* CAU45 (2384 ± 57) was 15.3 times higher than *B. cereus* DSMZ 4312 (156 ± 15) in HEp-2 cells (Figure 1c, *P* = 0.0003). Lastly, the production of cereulide in *B. cereus* CAU45 was quantified by UPLC-MS/MS assay. The amount of cereulide in *B. cereus* CAU45 (Figure 1e) was about 13.3 μg/mg biomass (wet weight) with the proportion of 93.8% in the crude extracts and it was seven times higher than in the reference strain *B. cereus* DSMZ 4312, which was about 1.9 μg/mg biomass (wet weight) (Figure 1d).

### 2.2. Quantification of Cereulide in Plasma by UPLC-MS/MS

**Figure 2:**
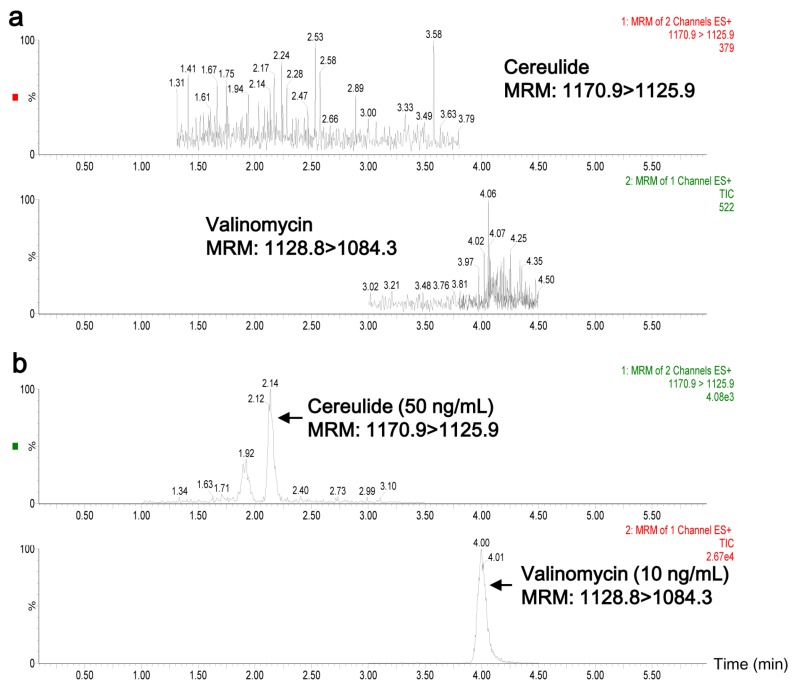
UPLC-MS/MS chromatograms of cereulide and valinomycin in rabbit plasma. (**a**) Blank plasma samples and (**b**) plasma sample spiked with extracted cereulide (50 ng/mL) from *B. cereus* CAU45 strain and valinomycin (10 ng/mL).

Representative chromatograms of blank plasma and plasma spiked with cereulide (50 ng/mL) and valinomycin (10 ng/mL) are shown in Figure 2. The retention times of cereulide and valinomycin were 2.14 min and 4.00 min. There were no interfering peaks at the retention times of targets of interest in the blank plasma (Figure 2a). The calibration curve of cereulide showed good linearity in the range from 1.5 ng/mL to 1000 ng/mL, with *r* = 0.9992. The limit of quantitation (LOQ, S/N = 10) was 1.5 ng/mL for cereulide in plasma. Additionally, the recoveries of cereulide spiked at three different concentrations (10, 50 and 100 ng/mL) were 98.7%, 87.8% and 104.4%, respectively. Together it indicated that the developed UPLC-MS/MS method in the present work was suitable for analyzing cereulide in plasma samples.

### 2.3. Toxicokinetics of Cereulide in Rabbits

**Figure 3:**
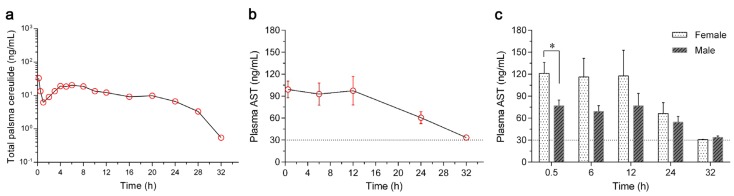
Concentrations of cereulide and aspartate aminotransferase (AST) in the plasma of rabbits. (**a**) A total number of eight rabbits (four female and four male) were used, with the weights of 2.0 ± 0.3 kg/rabbit. Cereulide extracted from the emetic *B. cereus* CAU45 strain was administrated through ear vein with the dose of 5 μg/rabbit, and plasma samples were collected at time points of 0, 0.2, 0.5, 1, 2, 3, 4, 5, 6, 8, 10, 12, 16, 20, 24, 28 and 32 h. Each plasma sample was spiked with valinomycin (10 ng/mL) as an internal standard to quantify cereulide by UPLC-MS/MS; (**b**) Concentrations of AST in the plasma were detected by EIA (Beyotime Biotechnology, Beijing, China), at the time points of 0.5, 6, 12, 24 and 32 h after a single intravenous injection of cereulide in rabbits. The concentrations of AST in plasma were below 30 μg/mL in negative controls, as indicated by the dash line. Means ± SD were presented throughout (*n* = 8); (**c**) Concentration dynamics of plasma AST in female and male rabbits. The AST concentrations of female rabbits were higher (*n* = 4,* *P* = 0.0345) than the males at the time point of 0.5 h, and other time points showed no significant difference.

A total number of eight rabbits (half female and half male) were used to evaluate the toxicokinetics of cereulide after a single administration of 5 μg cereulide. All plasma samples at different time points were collected, and the concentrations of cereulide were quantified by UPLC-MS/MS assay. The dynamic curve of cereulide in the plasma of rabbits is shown in Figure 3a, and toxicokinetic parameters were calculated using a non-compartmental model as shown in Table 1. Cereulide was detected in plasma within 0.2 h after a single intravenous injection and reached its maximum concentration of 40.8 ± 21.6 ng/mL. The concentration of cereulide dropped down until 1 h and rose thereafter. Following a short stationary phase from 4 h to 8 h, the concentration of cereulide decreased gradually. No cereulide could be detected 32 h later. Additionally, no cereulide was detected in the rabbits treated by 0.9% saline-methanol (10:90, *v*/*v*) based on our established UPLC-MS/MS method.

Liver damage in rabbits triggered by cereulide was evaluated by the concentration of aspartate aminotransferase (AST) in plasma. Comparing with the range of AST in the control (from 15.0 ng/mL to 30.0 ng/mL), a sharp increase in the AST concentration (99.1 ± 32.2 ng/mL) was found 0.5 h after the injection of cereulide and it lasted for 12 h (97.3 ± 55.3 ng/mL), as shown in Figure 3b. Liver function recovered, with the concentration of AST returning to the normal level 32 h after administration. In addition, the concentrations of AST in female rabbits were higher than those in males (Figure 3c), especially at the time point of 0.5 h (*P* = 0.0345).

## 3. Discussion

The increased incidence of *B. cereus* foodborne outbreaks is greatly associated with different types of foods [18,19,20,21,22]. Due to the formation of notorious endospores and the previously formed and heat-stable cereulide, emetic *B. cereus* can enter and survive during food production, processing and storage processes at various points, evoking vomiting in a few hours after ingestion [23]. Much more attention should be paid to the food matrices, especially the ones supporting the production of cereulide. Until now, emetic *B. cereus* group isolates have been widely reported in many countries [10,24,25], but only a few emetic *B. cereus* group strains were reported in China. For example, two emetic *B. cereus* strains and two emetic *B. weihenstephanensis* strains have been reported from fermented black beans and ice creams, respectively [12,19], and the prevalence of emetic *B. cereus* strains in these products remains unclear. In the present work, we firstly characterized an emetic *B. cereus* *sensu stricto* isolate from a local dairy farm of Beijing, China, with a high ability of cereulide production (Figure 1). Compared with the *ces* gene cluster of *B. cereus* plasmid pBCE4810 (GenBank accession No. NG_036207.1) [26], the *ces* sequence of *B. cereus* CAU45 showed a 99.9% identity. The production of cereulide can be also influenced by various factors [23], and further research is still needed to elucidate the enhanced production of cereulide. Additionally, the disability to express intact Nhe complex in *B. cereus* CAU45 makes it a great candidate to study the dissemination of emetic *B. cereus* and the function of cereulide in contaminated foods using animal models, without the influence of expressed enterotoxin Nhe as in most pathogenic *B. cereus* strains [27].

The toxicokinetics of extracted cereulide were evaluated in rabbits by the UPLC-MS/MS method. The concentration of cereulide in plasma rapidly decreased in the first hour after a single dose of intravenous injection (Figure 3a). Based on toxicokinetic analysis, we found that cereulide had a short elimination half-life (HL_Lambda_z) of 10.8 ± 9.1 h, with the mean retention time (MRTlast) of 9.6 ± 2.9 h and small volume of distribution (Vd) of 320.1 ± 399.6 mL, shown in Table 1. All data indicated that cereulide was eliminated or metabolized very fast in rabbits, which is consistent with the observation that the symptoms of the cereulide intoxication disappear after 6 h of food digestion [28].

The rapid increase of the AST concentration in plasma (Figure 3b) indicated the fast transfer of cereulide from blood to liver, resulting in liver injury with the liver losing its function. The time to recovery of the concentration of AST to normal range in rabbits was 32 h. AST reached its maximum concentration after 12 h of cereulide treatment in rabbits; however, it took 48 h to achieve the maximum concentration in mice [29]. Altogether, the different symptoms in various animal models might be ascribed to their physiological characters, or different doses of cereulide, or alternate routes of administration used in each animal model. In addition, higher levels of AST were also observed in female rabbits than male, indicating a gender difference may affect the metabolism of cereulide in rabbits as well.

In summary, we isolated a high virulent emetic *B. cereus* *sensu stricto* mutant strain, CAU45. This strain carried *nhe* and *ces* genes without the expression of intact Nhe complex. High production of cereulide in *B. cereus* CAU45 was correlated with its high cytotoxicity. Moreover, we studied the toxicokinetics of cereulide in rabbits after a single intravenous injection and monitored the damage in their livers. The toxicokinetic parameters might imply the rapid appearance of clinical symptoms. Interestingly, the toxic effect caused by cereulide showed a potential gender difference in rabbits. Altogether, the presence of emetic *B. cereus* in raw milk suggests that urgent sanitary practices are needed to eliminate emetic toxin and emetic *B. cereus*, both for improving the quality of milk and dairy products and reducing the incidence of *B. cereus*-associated diseases.

## 4. Materials and Methods

### 4.1. Bacterial Strains and Materials

*B. cereus* CAU45 was isolated from a raw milk sample in one local dairy farm of Beijing, China. *B. cereus* DSMZ 4312 was used as a reference strain for cereulide and Nhe, while *B. cereus* ATCC 14579 was chosen as a reference strain for Nhe and Hbl. All the monoclonal antibodies used for the detection of NheA and NheB, and CBP were as previously described [27]. HEp-2 cells used to evaluate the cytotoxicity of cereulide were obtained from Prof. Xiaojia Wang at China Agricultural University. Valinomycin purchased from Sigma-Aldrich (St. Louis, MO, USA) was used as an internal standard by UPLC-MS/MS test.

### 4.2. Isolation and Identification the Emetic B. cereus Strain

The isolation of *B. cereus* was performed according to the previous protocol [30]. Briefly, the blue/green colonies on Brilliance™ *Bacillus cereus* Agar (Oxoid, Basingstoke, UK) plates, after incubating at 32 °C for 24 h, were selected as presumptive *B. cereus* strains. The positive colonies were subsequently passaged on brain heart infusion (BHI, Land Bridge Technology, Beijing, China) agar plates, and single colonies were chosen for the further study.

The genomic DNA was extracted by boiled water method and the plasmid was extracted by QIAfilter Plasmid Midi Kits (QIAGEN, Duesseldorf, Germany) as described before [31]. Four main toxin genes, *nhe*, *hbl*, *cytK1* and *ces*, were tested by PCR assays [32]. The amplicons of targets were sequenced and blasted with reference sequences by DNAMAN 8.0.5.789 software (Lynnon Biosoft, San Ramon, CA, USA). Caseinhydrolysat-glucose-yeast (CGY) medium plus 1% glucose (Sigma-Aldrich, St. Louis, MO, USA) was used to produce Nhe [27]. The presence of Nhe and CBP was detected by enzyme immunoassays (EIAs) as previously described [27,33]. HEp-2 cells were used to evaluate the cytotoxicity of cereulide based on water-soluble tetrazolium salt-1 (WST-1, Roche, Basel, Switzerland) method [34].

### 4.3. Extraction of Cereulide

Cereulide was extracted from the cultures of *B. cereus* CAU45 and DSMZ 4312 on trypticase soy agar (TSA, Land Bridge Technology, Beijing, China) plates at 32 °C for 10 days. Briefly, bacterial cultures were collected and extracted with methanol (approximate 100 mg bacterial biomass, wet weight dissolved in 1 mL methanol, Fisher Scientific, Waltham, PA, USA), heated at 80 °C for 30 min. The residues were resuspended in 0.5 mL methanol, vortexed for 2 min and centrifuged at 13,000 *g* for 5 min. The supernatants were evaporated to dryness with a flow of nitrogen gas, and dissolved in methanol. All the extracts were stored in dark glass vials at −20 °C.

### 4.4. Cereulide Treated Rabbits

This animal study was approved by the Institutional Animal Care and Use Committee at the China Agricultural University (SYXK (2012-0037), 28 December 2012), and all the operations were conducted according to the biosafety procedures. Eight adult New Zealand White rabbits (four female and four male) with weights of 2.0 ± 0.3 kg were used in this study. A single intravenous dose of 5 μg cereulide dissolved in 0.9% saline-methanol (10:90, *v*/*v*; the amount of methanol injected to rabbit was about 1.8 g/kg body weight) was administrated. Two other rabbits (one female and one male) were treated by 0.9% saline-methanol (10:90, *v*/*v*) as control. Blood samples were collected after injection at different time points 0, 0.2, 0.5, 1, 2, 3, 4, 5, 6, 8, 10, 12, 16, 20, 24, 28, 32 h. All samples were centrifuged at 4 °C, 2000 *g* for 10 min. Plasma from each sample was collected and stored at −80 °C.

### 4.5. Quantification of Cereulide in Plasma by UPLC-MS/MS

The plasma samples were thawed to room temperature before analysis. Then 200 μL of each sample was spiked with 10 ng/mL valinomycin (Sigma-Aldrich, St. Louis, MO, USA) as internal standards in a 1.5 mL tube (Axygen, Union City, CA, USA). The extraction steps were as below. First 1 mL acetonitrile (Fisher Scientific, Waltham, PA, USA) was added to each sample and vortexed for 3 min. After centrifugation at 13,000 *g* for 10 min, the supernatants were transferred into new tubes. The extraction steps were repeated once and the supernatants were combined. The supernatants were evaporated under a flow of nitrogen gas at 50 °C. The dry samples were reconstituted with 500 μL methanol and water with the ratio of 9:1 (*v*/*v*), vortexed for 3 min and filtered through 0.22 μm filter membrane (PALL, Port Washington, NY, USA) before UPLC-MS/MS analysis.

The presence of cereulide was confirmed in all extracts and quantified by UPLC-MS/MS as described before [35]. UPLC-MS/MS (Waters, Milford, MA, USA) equipped with a RP C18 column (50 mm × 2.1 mm, 1.7 μm, Acquity UPLC, Milford, MA, USA) was used. A gradient program was applied with the mobile phase consisting of solvent A (0.1% formic acid (Dikma, Lake Forest, CA, USA) in water) and solvent B (0.1% formic acid in acetonitrile) as follows: 98%–10% A (0–1 min), 10%–0% A (1–3 min), 0%–98% A (3–5 min). The flow rate was set at 0.3 mL/min with the injection volume of 10 μL. A positive electrospray ionization (ESI^+^) mode was chosen to analyze the transitions of *m*/*z* 1170.9 → 1125.9 for cereulide and *m*/*z* 1128.8 → 1084.3 for valinomycin, by multiple reaction monitoring (MRM) method. The cone voltages for cereulide and valinomycin were 10 V and 60 V, and collision energy were 20 eV and 37 eV, respectively. All data were acquired and processed by software Masslynx 4.1 (Waters, Milford, MA, USA, 2005). The toxicokinetic parameters of cereulide were analyzed by the commercial pharmacokinetic program Phoenix WinNonlin 5.2.1 (Certara, Princeton, NJ, USA).

### 4.6. AST Measurement

Plasma samples at time points of 0.5, 6, 12, 24 and 32 h were evaluated concentrations of AST by indirect EIA according to the instruction (Beyotime Biotechnology, Beijing, China). A five-fold dilution of each plasma sample with phosphate buffer saline (PBS, pH = 7.4) was coated in a microtiter plate (Corning, Corning, NY, USA) at 37 °C for 2 h. The primary antibody and the secondary antibody were added in turn with incubation at 37 °C for 1 h. Subsequently, the substrate was added with the absorbance at 450 nm.

## Figures and Tables

**Table 1:** Toxicokinetic parameters of a single intravenous injection of cereulide in rabbits.

Toxicokinetic Parameters *	Median	Range	Mean ± SD
AUC (h × ng/mL)	309.3	81.2–851.1	320.9 ± 240.1
AUC_∞_ (h × ng/mL)	330.5	126.0–853.9	390.3 ± 257.9
Cl (mL/h)	15.5	5.9–39.7	19.1 ± 12.8
C_max_ (ng/mL)	33.8	13.3–78.1	40.8 ± 21.6
HL_Lambda_z (h)	6.9	1.9–24.5	10.8 ± 9.1
Lambda_z (1/h)	0.1	0.03–0.4	0.1 ± 0.1
MRTlast (h)	10.6	4.5–13.4	9.6 ± 2.9
T_max_ (h)	0.2	0.2–8.0	2.6 ± 3.4
Vd (mL)	208.1	16.3–1254.5	320.1 ± 399.6

* The toxicokinetic parameters were analyzed by the commercial pharmacokinetic program Phoenix WinNonlin 5.2.1 (Certara). AUC, area under the curve for the plasma concentration until the last time point; AUC_∞_, area under the curve until infinity; Cl, clearance; C_max_, maximum concentration after intravenous administration; HL_Lambda_z, elimination half-life; Lambda_z, elimination rate constant; MRTlast, mean retention time until the last time point; T_max_, time to peak concentration; Vd, volume of distribution.
